# Development and applications of metabolic models in plant multi-omics research

**DOI:** 10.3389/fpls.2024.1361183

**Published:** 2024-10-17

**Authors:** Yonggang Gao, Cheng Zhao

**Affiliations:** Shenzhen Branch, Guangdong Laboratory of Lingnan Modern Agriculture, Key Laboratory of Synthetic Biology, Ministry of Agriculture and Rural Affairs, Agricultural Genomics Institute, Chinese Academy of Agricultural Sciences, Shenzhen, China

**Keywords:** plants, multiomics, metabolic models, metabolic networks, development and challenges

## Abstract

Plant growth and development are characterized by systematic and continuous processes, each involving intricate metabolic coordination mechanisms. Mathematical models are essential tools for investigating plant growth and development, metabolic regulation networks, and growth patterns across different stages. These models offer insights into secondary metabolism patterns in plants and the roles of metabolites. The proliferation of data related to plant genomics, transcriptomics, proteomics, and metabolomics in the last decade has underscored the growing importance of mathematical modeling in this field. This review aims to elucidate the principles and types of metabolic models employed in studying plant secondary metabolism, their strengths, and limitations. Furthermore, the application of mathematical models in various plant systems biology subfields will be discussed. Lastly, the review will outline how mathematical models can be harnessed to address research questions in this context.

## 1 Introduction

As plants are immobile organisms, they must possess the ability to conform to ever-changing surroundings in order to survive, thrive, and complete their life cycles, utilizing intricate physiological processes. Plant-derived compounds hold substantial potential for sustainable advancement. Over the last decade, plant genomics research has progressed rapidly, integrating methodologies such as whole-genome sequencing, comprehensive transcriptome analysis, single-cell sequencing, spatial transcriptomics, spatial metabolomics, and comparative genomics sequencing (Daloso and Williams, 2023). Decreased data generation and analysis costs have led to a significant rise in the production and scrutiny of multiomics data within a brief duration (Roy et al., 2021). Consequently, adjustments to the construction techniques of mathematical models are essential to fulfill heightened application prerequisites (Wang et al., 2019a).

Amidst the persistent expansion of the global populace, contemporary agricultural practices are compelled to ensure the stability and security of food resources (Li et al., 2019; Reynolds et al., 2021). The fusion of genomics, transcriptomics, and metabolomics presents significant potential for enriching our comprehension of intricate crop characteristics and elucidating the genetic pathways governing essential phenotypes (Reynolds et al., 2021; Moreira et al., 2020). The utilization of mathematical models to delineate the genetic makeup of plants, their metabolites, and the diversity of observable traits is of paramount significance.

Metabolic modeling has emerged as a pivotal tool for steering metabolic engineering endeavors (Morgan et al., 2002; Moreira et al., 2019; Shaw and Cheung, 2021), particularly in the optimization of chemical production in microbial organisms. Furthermore, it facilitates the direct and sustainable extraction of numerous bioactive compounds from plants (Nakamasu et al., 2019). The plant science community is increasingly recognizing the benefits of metabolic modeling for metabolic engineering and systems biology (Smithers et al., 2019; Shaw and Cheung, 2021).

**Figure 1:**
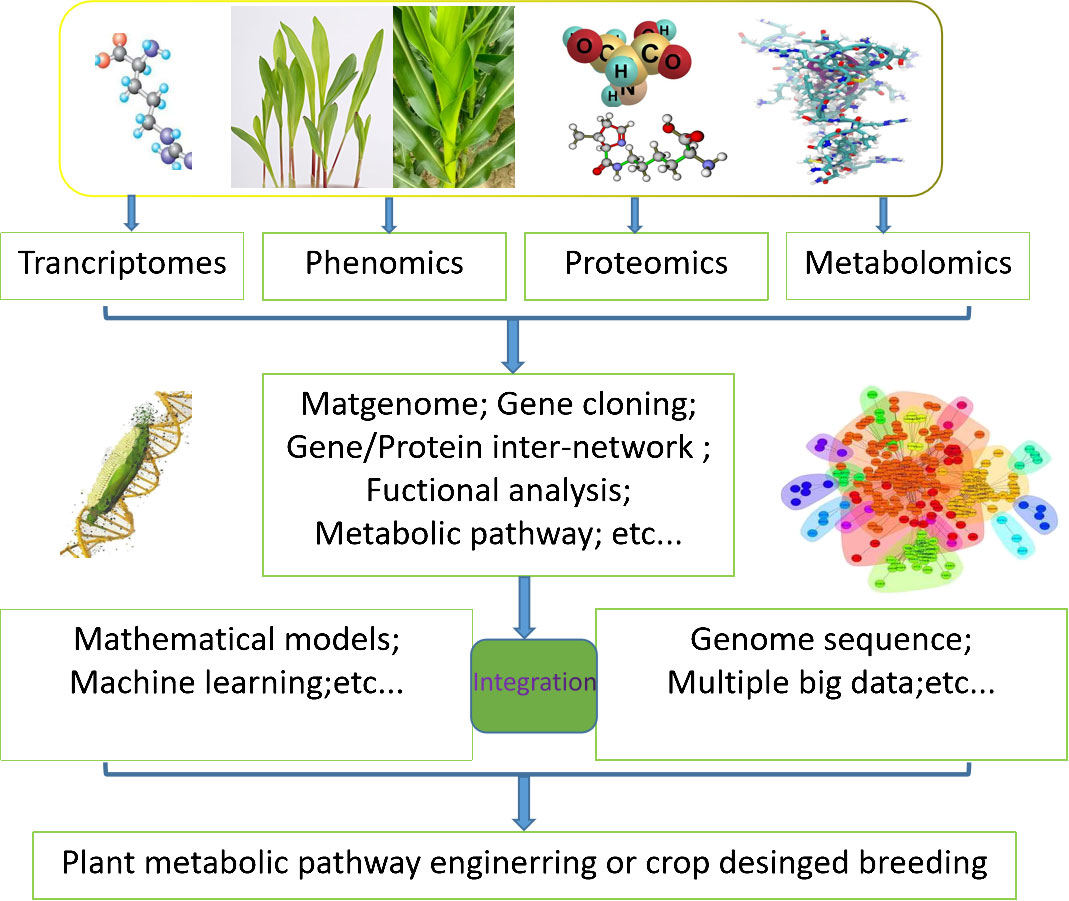
Application pattern diagram of plant systems biology research and the combination of metabolic models and multiomics data. The foundational concept delineated in this framework underscores the extensive utilization of transcriptome, phenome, proteome and metabolome datasets within the realm of plant biology investigation.The amalgamation of multi-omics data through the employment of mathematical models, machine learning, and other bioinformatics methodologies can elucidate the evolutionary and adaptive processes of plants within distinct ecological niches. Factors such as genomic structural variance, gene-protein interactions, gene functionality scrutiny, cellular signal transduction cascades, and system biology insights pertaining to growth and metabolic control are vital elements of this genetic information landscape, serving as crucial guidelines for the advancement of plant metabolic engineering and the enhancement of crop breeding strategies.

Metabolic models have been widely applied in microorganism metabolic engineering for the production of biofuels, amino acids, and other bioproducts, as well as in medical research to investigate cancer metabolism, antimicrobial target identification, and personalized drug therapy (Guijas et al., 2018). Due to the limited knowledge and intricate nature of plant metabolism, it is still challenging to understand plant metabolic networks and apply those understandings to plant metabolic engineering (Chalmandrier et al., 2021). The integration of metabolic models and multi-omics data can facilitate a systematic, continuous, and precise analysis of diverse plant processes, encompassing dynamic growth, environmental impacts, and coordination of secondary metabolism, among others (**Figure 1**). In this review, we mainly focus on recent the development of constraint-based modeling and kinetic modeling for plant researches. Methods of multi-omics data integration into metabolic models are emphasized, whereas the dilemmas and challenges are discuss in this review.

## 2 Types of mathematical models for plants

Among various modeling approaches, metabolic pathway analysis has the capability to delineate the fundamental functional modes of different core metabolism subsections, such as photorespiration (Jendoubi et al., 2020). Furthermore, it can unveil the interconnected functionality observed in classical metabolic pathway definitions (Moreira et al., 2019; Lacchini and Goossens, 2020). Currently, a wide array of mathematical tools are available for analyzing biological processes in plants, encompassing large-scale genomes and metabolic networks, and have been extensively utilized in studies employing constraint modeling.

The mathematical model employed in plant metabolic research include: constraint-based models (CBMs) (also called flux balance analysis (FBA) models, with subtypes of genome-scale metabolic (GEMs) models and proteome-constrained (PCMs) models) and enzyme kinetic (EKMs) models. These systems biology tools are utilized to investigate the intricacies of biological metabolic networks (Nagegowda et al., 2020). Notably, FBA is the most extensively applied constraint-based approach, leveraging linear programming to forecast the distribution of metabolic fluxes throughout the network. In contrast to enzyme kinetic models that require extensive kinetic parameter data to capture the dynamic behavior of metabolic processes, constraint-based models focus on the overall network characteristics without necessitating precise kinetic information. FBA typically assumes a steady-state condition with no metabolite accumulation, and optimizes an objective function, such as maximizing growth rate (Shaw et al., 2021). Genome-scale metabolic models are comprehensive computational representations of the metabolic capabilities of an organism, constructed based on the organism's genomic information (Antonakoudis et al., 2020). These models typically encompass hundreds to thousands of metabolic reactions, enabling the application of FBA to study the metabolic characteristics of the entire organism (Moreira et al., 2019). In contrast, enzyme kinetic models focus on describing the dynamic behavior of individual enzymatic reactions, such as through the utilization of the michaelis-menten equation, to capture the kinetics of specific metabolic pathways (Leow et al., 2019). Genome-scale metabolic models provide the foundational framework for constraint-based modeling approaches, while proteome-constrained models represent an extension of GEMs that incorporate proteomic constraints (Noshita et al., 2020). This allows PCMs to more accurately capture the impact of protein allocation on metabolic fluxes, providing a deeper understanding of the interplay between an organism's proteome and its metabolic network. The progression from enzyme kinetic models to genome-scale metabolic models, and then to proteome-constrained models reflects an increasing level of integration, moving from the modeling of individual enzymatic reactions to the comprehensive representation of the entire metabolic network, and further incorporating proteomic constraints to enhance the accuracy and predictive power of metabolic simulations. In this summary, we will systematically review these models.

### 2.1 Constraint-based model

A Constraint-Based Model (CBM) functions as an analytical construct integrating diverse constraints to demarcate the operational boundaries of a designated system. These constraints originate from various origins such as established physical principles, empirical evidence, and theoretical constructs. Serving as a prognostic instrument, the CBM anticipates the system's behaviors and adjustments under varied circumstances, while maintaining adherence to the stipulated constraints (Shaw et al., 2021). Through resolving these constraint conditions, CBM is capable of pinpointing an optimal solution or a range of feasible solutions that satisfy all the constraints (Antoniewicz et al., 2015; Colombié et al., 2017; Groot et al., 2020).

CBMs have been utilized in investigating and modulating the metabolic pathways related to plant growth, development, and response to environmental stresses. Through the application of CBMs, researchers can pinpoint crucial metabolic reactions and potential genetic targets for enhancement of crop yield, stress tolerance, and nutritional quality (Varshney et al., 2018; Groot et al., 2020). Additionally, CBMs have been employed to optimize metabolic pathways for biofuel production from plants. By simulating and scrutinizing plant metabolic networks, researchers can pinpoint potential targets for genetic manipulation aimed at improving the production of biofuel precursors, such as sugars, lipids, and biobased chemicals (Rong et al., 2021). Furthermore, CBMs have been utilized in the study of biosynthesis pathways of phytochemicals, which have pharmaceutical or nutritional significance as secondary metabolites in plants. Through the analysis of metabolic networks, researchers can identify potential genetic targets or optimal culture conditions to enhance the production of specific compounds.

Despite its usefulness in plant metabolism research, CBM models have a number of drawbacks. One such limitation is the substantial requirement of computational resources and time, particularly when dealing with large-scale metabolic networks found within intricate plant cells. Moreover, CBM heavily relies on precise parameters, such as metabolic reaction rates and metabolite concentrations, which can be challenging to measure or estimate accurately (Martin et al., 2016; Heirendt et al., 2019). Additionally, CBM assumes that chemical reactions in the metabolic network have reached a steady state, consequently neglecting nonequilibrium conditions and dynamic changes that may occur. In conclusion, although CBM enables systematic analysis and prediction of plant metabolism, it is accompanied by computational complexities and limitations in parameterization.

### 2.2 Enzyme kinetic models

Enzyme kinetic models serve as valuable tools in guiding enzyme engineering endeavors directed towards enhancing catalytic efficiency, substrate specificity, and enzyme stability. These models play a pivotal role in formulating rational mutations and strategies for directed evolution to boost enzyme functionality (Martins et al., 2016; Nagegowda et al., 2020). Research has utilized kinetic models to investigate the biosynthesis of B2 in rice, with predictions indicating that OsRibA serves as a rate-limiting enzyme in this pathway. Subsequent experiments have shown that overexpression of the OsRibA gene leads to a significant increase in riboflavin production. The kinetic model, while advantageous, presents limitations in plant metabolism research. This encompasses the complexity resulting from the comprehensive modeling of the entire plant metabolic system which involves numerous metabolic pathways and reactions (Tenenboim et al., 2016). Moreover, the establishment of accurate dynamic models necessitates a substantial amount of experimental data for the evaluation of metabolic pathway parameters, consuming significant time and resources (Chalmandrier et al., 2021). Additionally, the model's accuracy may be influenced by diverse environmental factors and conditions affecting plant metabolic processes, particularly within complex growth environments (Colombiéet al., 2017; Dale et al., 2021). Although the kinetic model offers advantages in plant metabolism research, challenges arise regarding modeling complexity and parameter estimation, requiring a comprehensive examination of these factors to fully harness its potential.

Enzyme kinetic models are extensively utilized in plant science to investigate the dynamics of enzyme-catalyzed reactions and to gain insights into the control of plant metabolism. They have been applied to analyze the kinetics of pivotal enzymes within photosynthetic pathways, such as RuBisCO and ATP synthase. Such models facilitate comprehension of the determinants affecting photosynthetic efficacy and pinpointing potential avenues for enhancing crop yield (Chen et al., 2022). Enzyme kinetic models serve as valuable tools in guiding enzyme engineering endeavors directed towards enhancing catalytic efficiency, substrate specificity, and enzyme stability. These models play a pivotal role in formulating rational mutations and strategies for directed evolution to boost enzyme functionality (Martins et al., 2016; Nagegowda et al., 2020). Research has utilized kinetic models to investigate the biosynthesis of B2 in rice, with predictions indicating that OsRibA serves as a rate-limiting enzyme in this pathway. Subsequent experiments have shown that overexpression of the OsRibA gene leads to a significant increase in riboflavin production. The kinetic model, while advantageous, presents limitations in plant metabolism research. This encompasses the complexity resulting from the comprehensive modeling of the entire plant metabolic system which involves numerous metabolic pathways and reactions (Tenenboim et al., 2016). Moreover, the establishment of accurate dynamic models necessitates a substantial amount of experimental data for the evaluation of metabolic pathway parameters, consuming significant time and resources (Chalmandrier et al., 2021). Additionally, the model's accuracy may be influenced by diverse environmental factors and conditions affecting plant metabolic processes, particularly within complex growth environments (Colombiéet al., 2017; Dale et al., 2021). Although the kinetic model offers advantages in plant metabolism research, challenges arise regarding modeling complexity and parameter estimation, requiring a comprehensive examination of these factors to fully harness its potential.

### 2.3 Genome-scale metabolic models

Genome-Scale Metabolic Models (GEMs) are comprehensive computational frameworks that encompass the entirety of an organism's metabolic network, derived from its genomic information. These models are meticulously constructed using the genetic information encoded in an organism's DNA, offering a detailed representation of the complex metabolic pathways and reactions that support cellular processes. GEMs are constraint-based models that function as computational depictions of an organism's metabolism, integrating genomic sequences, biochemical pathways, and experimental data to predict its metabolic capabilities (Aurich et al., 2016; Antonakoudis et al., 2020). Generation of GEMs predominantly relies on genome annotation data for gene identification and functional categorization within an organism’s genetic makeup. Once established, GEMs can be leveraged for simulating diverse metabolic phenomena such as growth rates, nutrient assimilation, and metabolite synthesis (Chen et al., 2020).

GEMs are typically conceptualized as constraint-based models comprising a complex system of mathematical equations, defining the stoichiometry, thermodynamics, and regulatory constraints of the metabolic network. Employing mathematical optimization algorithms enables GEMs to project optimal flux distributions, serving to maximize specific objectives, such as biomass production or ATP yield. Recently, there has been significant development in GEMs that integrate genomic, transcriptomic, proteomic, and thermodynamic data (Botero et al., 2018; Clark and Donoghue, 2018; Bogaert and Myers, 2019; Noshita et al., 2022). Several software programs, such as CarveMe, Path2Models, ModelSEED, AGORA, REVEN 2.0, and SuBliMi NALToolbox, have been developed to facilitate the reconstruction of genome-scale metabolic models (GEMs). These tools are capable of automating tasks such as genome annotation, gene-protein-reaction (GPR) association generation, and predicting reaction reversibility (Seaver et al., 2014; Villegas et al., 2017; Arya et al., 2020).

GEMs require genome scale enzyme annotation data from Pathway/Genome database such as PlantCyc and KEGG (Schläpfer et al., 2017; Zhan et al., 2022). Several software programs, such as COBRA toolbox, CarveMe, Path2Models, ModelSEED, AGORA, REVEN2.0, and SuBliMi naLToolbox, have been developed to facilitate the reconstruction of genome-scale metabolic models (GEMs). These tools are capable of automating tasks such as genome annotation, gene-protein-reaction (GPR) association generation, and predicting reaction reversibility (Seaver et al., 2014; Villegas et al., 2017; Heirendt et al., 2019; Nagegowda et al., 2020).

Despite their advantages, GEMs also present certain limitations. The accurate establishment of GEMs necessitates the acquisition of complete sequence information regarding the plant genome, extensive data integration, and meticulous model construction, posing challenges for genotypes that are incomplete or atypical. Furthermore, the validation of GEMs’ predictive outcomes through experimental verification can be intricate and time-consuming. Additionally, precise experimental data on model parameters such as metabolic rea ction rates and metabolite concentrations are essential for GEM development. GEMs commonly assume steady-state conditions in metabolic networks, although plant metabolism can exhibit dynamic behavior across various growth stages, environmental settings, and stress conditions, thereby limiting the broad applicability of GEMs.

### 2.4 Proteome-constrained models

The proteome-constrained model represents a computational framework that incorporates constraints derived from the proteome, encompassing the entire complement of proteins synthesized by a genome under specified conditions. This model employs constraints informed by experimental data, including protein concentrations, enzyme kinetics, and protein-protein interactions. It serves as a valuable tool for analyzing and predicting the dynamics of cellular systems concerning protein expression and functionality. Proteome-constrained models play a pivotal role in the systems-level investigation of cellular processes (Bellasio et al., 2018; Lu et al., 2022), enhancing our comprehension of intricate biological systems, and facilitating the development of therapeutic interventions (Courdavault et al., 2021). The proteome-constrained modeling approach leverages knowledge of an organism's proteome to integrate genetic information, encompassing genes and proteins, into mathematical frameworks for simulating and studying cellular metabolism. In this modeling paradigm, the metabolic network is represented as a set of interconnected biochemical reactions governed by metabolite fluxes. Each reaction is associated with the proteins that facilitate catalysis, and their quantitative levels or expression profiles serve as regulatory constraints. These essential data are typically derived from experimental methodologies such as mass spectrometry or RNA sequencing (Chen et al., 2021; Lu et al., 2022).

Protein-constrained models that incorporate proteomics data provide more accurate predictions of cellular functionality compared to conventional metabolic models based solely on genome-scale metabolic reconstruction. This is due to the fact that proteomics data provides information on protein expression levels, post-translational modifications, and protein-protein interactions, which are essential for understanding cellular function. By integrating these data, protein-constrained models can provide a more comprehensive description of cellular metabolism, leading to more accurate predictions (Wang et al., 2019b). These models facilitate the anticipation of metabolic flux patterns, detection of metabolic limitations, and examination of relationships among genes, proteins, and reactions. Consequently, methodologies like flux balance analysis (FBA) or its modifications have been utilized to scrutinize the model and prognosticate cellular behavior under diverse circumstances.

The metabolic flux in networks is influenced by various additional constraints (Janasch and Asplund-Samuelsson, 2018). The stoichiometric aspect of GEM metabolic networks has limitations, including regulation through gene expression, posttranslational modifications, and enzyme characteristics determined by protein structure (Chen and Nielsen, 2019; Dong et al., 2022). By integrating cellular processes and protein structure information into the model, comprehensive multiomics data analysis can be performed (Jendoubi and Ebbels, 2020). This will allow the model to achieve a deeper understanding of the fundamental principles that govern complex cellular metabolic regulation and evolution. Therefore, large-scale multiscale whole-cell models are urgently needed.

## 3 Mathematical models have emerged as invaluable tools in plant research

Mathematical models have become essential instruments in the domain of plant investigation, providing significant insights and prognostications. By employing mathematical equations, investigators can depict and measure various phenomena pertaining to plants (Tokuda et al., 2022). These models, furnishing indispensable tools and insights, occupy a central position in the progression of plant research (Pouvreau et al., 2018; Shafiee-Gol et al., 2021).

Plant genomics, transcriptomics, proteomics, metabolomics, single-cell transcriptome and other omics technologies have rapidly developed in the past decade, leading to an explosion of sequenced genomes, as observed by plant biologists (Lu et al., 2022). Genome sequences serve as fundamental units for constructing functional plants. Consequently, molecular plant biologists encounter the challenge of comprehending the combined functionality of tens of thousands of genes encoded in each genome (Wang et al., 2019a). The intricate regulatory networks established by these genes contribute to consistent growth and developmental patterns under different environmental conditions (Varshney et al., 2018; Bogaert et al., 2019; Zhan et al., 2022). The effective integration of multiomics data for analyzing genome-scale metabolic models in the era of big data is crucial. Achieving a comprehensive understanding and accurate prediction of how omics data and gene/metabolite networks ultimately regulate growth and development is an immense challenge, if not impossible (Razzaque et al., 2019; Dong et al., 2022).

### 3.1 Plant growth and development

To achieve a comprehensive understanding of the molecular mechanisms underlying all aspects of plant biology, it is necessary to employ a comprehensive set of models. Each model should have the ability to assess at least one aspect of plant life. Wang (2016) integrated a genome-scale metabolic flux model with transcriptomic data to investigate the metabolic reactions of Arabidopsis thaliana under both low and high CO2 conditions. However, the utilization of transcriptomic data alone often fails to produce the anticipated enhancement in model prediction. Benes (2020) demonstrated with their multiscale model that the increased leaf production rate in transgenic Arabidopsis with aberrant developmental regulation is large enough to account for the smaller leaf phenotype in this genetically modified plant. The output of the clock submodule is utilized to regulate tissue elongation and starch metabolism. With these updates, Arabidopsis FMv2 is capable of predicting the phenotypic response to changesin circadian rhythm caused by clock mutations in plants. Heirendt (2019) introduced an updated version of the COBRA Toolbox, specifically the COBRA Toolbox v3.0. This version incorporates novel techniques for quality control reconstruction, modeling, topological analysis, strain and experimental design, and network visualization. Additionally, it enables the integration of chemical informatics, metabolomics, transcriptomics, proteomics, and other data types into networks. Plant photosynthetic metabolism The distribution of photosynthetic products in plants is a complex process influenced by a variety of factors, including environmentalelements such as light, water, and temperature, as well as the plant's own genetic characteristics and growth development. Research on plant photosyntheticmetabolism models is a complex and ever-evolving field that can deepen our understanding of how plants convert light energy into chemical energy through photosynthesis. To support studies of photosynthetic nitrogen assimilation and its complex interaction withphotosynthetic carbon metabolism for crop improvement, we developed a dynamic systems model of plant primary metabolism, which includes the Calvin-Benson cycle, the photorespiration pathway, starch synthesis, glycolysis-gluconeogenesis, the tricarboxylicacid cycle, and chloroplastic nitrogen assimilation. This model successfully captures responses of net photosynthetic CO2 uptake rate (A), respiration rate, and nitrogen assimilation rate to different irradiance and CO2 levels. Examines how photosynthesis in Arabidopsis thaliana acclimates to cold temperatures,The study suggests that the ability to acclimate photosynthesis to environmental changes, including cold, is important for plant fitness and seed yield, which could have implications for crop breeding and agricultural practices.The research employs metabolic models to show how the relative export of triose phosphate and 3-phosphoglycerate from the chloroplast could provide a signal of the chloroplast redox state, potentially underlying the photosynthetic acclimation to cold. Integrates relative gene expression levels from multiple transcriptomic and proteomic datasets into flux balance analysis(FBA) predictions for a multi-tissue model of Arabidopsis thaliana's central metabolism.Plant-microbe interactionsPlant-microbe-environment interaction modeling is an important branch at the intersection of ecology, microbiology, and botany, focusing on the interplay between plants, microbes, and their environment. These interaction models help us understand how plants adapt to environmental changes, improve nutrient absorption efficiency, enhance disease resistance, and influence thefunctioning of ecosystems through their interactions with microbes. Sun (2022) explores the metabolic interactions between the inoculant bacterium bacillus velezensis SQR9. Metabolic modeling and profiling were used to demonstrate metabolic facilitation between the bacterial strains, suggesting a form of cross-feeding that enhances communityperformance. Found a strong phylogenetic signature in the carbon source utilization profiles of the strains. The genome-scale models provided further insight,correctly predicting positive outcomes and emphasizing the role of carbon metabolism in community assembly. The present plant models discussed in this study signify an advancement in our endeavor to investigate and understand the various plant forms and functions. Nevertheless, there are enduring challenges in the domains of comprehensive growth models, integration of large-scale models, maintaining equilibrium between growth and metabolism, and the precision of model forecasts. The ongoing progression of innovative technologies in molecular biology and bioinformatics is already facilitating the creation of the upcoming plant models.Metabolic phenotypes are primarily defined bythe levels of metabolites, which are established by a complex network of interrelated biochemical reactions in genome-scale metabolic networks. To better understand these systems, several genome-scale metabolic reconstructions have recentlybeen published for plant species (Seaver et al., 2014). The methods employed to study these metabolic models, such as flux balance analysis (FBA), considerall reactions in the model when attempting to predict a biological phenotype, such as plant growth. Here, we summarize the currently available design software and R packages used in genome-scale metabolic networks (**Table 1**).

**Table 1:** Bioinformatics tools for plant metabolomics workflow.

Tool	Weblink	Major Function
MetaboAnalyst	www.metaboanalyst.ca/	Statistical analysis
MeltDB 2.0	https://meltdb.cebitec.uni-bielefeld.de	Data processing
MetaP-server	http://metabolomics.helmholtz-muenchen.de/metap2/	Data analysis
MetExplore	http://metexplore.toulouse.inra.fr	Pathway analysis
Metabox	https://github.com/kwanjeeraw/metabox	Analysis workflow
METLIN	https://metlin.scripps.edu/	Metabolite annotation
MetAlign	www.metalign.nl	Workflow analysis
MetaboAnalystR	https://github.com/xialab/MetaboAnalystR	R package
Lilikoi	https://github.com/lanagarmire/lilikoi	R package
MetFrag	http://c-ruttkies.github.io/MetFrag	Metabolite annotation
MetaGeneAlyse	http://metagenealyse.mpimp-golm.mpg.de/	Metabolite data analysis
Metacrop 2.0	http://metacrop.ipk-gatersleben.de	Data annotation
MetAssign	http://mzmatch.sourceforge.net/	Data annotation
MET-COFEA	http://bioinfo.noble.org/manuscript-support/met-cofea/	Data processing
MetPA	http://metpa.metabolomics.ca	Pathway analysis
iMet-Q	http://ms.iis.sinica.edu.tw/comics/Software_iMet-Q.html	Data processing
Babelomics 5.0	http://www.babelomics.org/	Statistical analysis
XCMS	https://xcmsonline.scripps.edu	Data processing
MZedDB	http://maltese.dbs.aber.ac.uk:8888/hrmet/index.html	Data annotation
MassBank	http://www.massbank.jp/	Metabolite annotation
MaxQuant	https://www.maxquant.org/	Data annotation & processing
MetFusion	http://mgerlich.github.io/MetFusion/	Integrated compound detection
MAVEN	https://maven.apache.org/	Data processing
MZmine2	http://mzmine.github.io/	Data processing
MSEA	http://www.metaboanalyst.ca/	Pathway analysis
MS-Dial	http://prime.psc.riken.jp/Metabolomics_Software/MS-DIAL/	Data processing
MarVis	http://marvis.gobics.de/	Metabolite annotation
Mummichog	http://mummichog.org	Pathway analysis
MMCD	http://mmcd.nmrfam.wisc.edu/	Metabolite annotation
COVAIN	http://www.univie.ac.at/mosys/software.html	Statistical analysis
CAMERA	https://bioconductor.org/packages/release/bioc/html/CAMERA.html	Data annotation
CFM-ID	http://cfmid.wishartlab.com	Metabolite identification
ADAP	http://www.du-lab.org/software.htm/	Data processing
KEGG	http://www.genome.jp/kegg/	Metabolic models
GenePattern	http://software.broadinstitute.org/cancer/software/genepattern/	Statistical analysis
Galaxy-M	https://github.com/Viant-Metabolomics/Galaxy-M	Workflow analysis
RetSynth	https://github.com/sandialabs/RetSynth	Workflow analysis
CNApy	https://github.com/cnapy-org	Workflow analysis
OptFlux	http://www.optflux.org	Workflow analysis
COBRA Toolbox	(https://github.com/opencobra/cobratoolbox	Workflow analysis
FastMM	https://github.com/GonghuaLi/FastMM	Workflow analysis
Medusa	https://github.com/opencobra/Medusa	Workflow analysis
Ssbio	http://github.com/SBRG/ssbio	Workflow analysis
Mackinac	https://github.com/mmundy42/mackinac	Workflow analysis
Pybel	https://github.com/pybel	Workflow analysis
COBRApy	http://opencobra.sourceforge.net	Workflow analysis
PyscesToolbox	https://github.com/PySCeS/PyscesToolbox	Workflow analysis
MEMOTE	https://memote.io	Workflow analysis
DORMAN	http://ciceklab.cs.bilkent.edu.tr/dorman	Workflow analysis
GPRuler	https://github.com/qLSLab/GPRuler	Workflow analysis
Diurnal.plant.tools	http://diurnal.plant.tools	Workflow analysis
Plant seed	http://plantseed.theseed.org	Workflow analysis
BiGG Models	http://bigg.ucsd.edu	Workflow analysis
MetaNetX	http://www.metanetx.org	Workflow analysis
Model SEED	http://modelseed.org	Workflow analysis

## 4 Challenges and dilemmas faced by mathematical models in plant research

High-throughput experiments that analyze genomes, transcriptomes, proteomes, and metabolomes generate a large quantity of concurrently measured molecular entities (Tenenboim and Brotman, 2016). In current biological research, a combination of experimental high-throughput techniques is often used to investigate a broad range of complex research questions (Seaver et al., 2014; Zhou et al., 2021). High-throughput sequencing (HTS) technologies have revolutionized genetics and genomics at the genome level, providing comprehensive information on the genomes of numerous species through sequencing projects (Arya et al., 2020; Moreira et al., 2020; Sun et al., 2021).

However, the integration of big data encounters numerous challenges, including issues related to data quality and consistency, the management of large and intricate data sets, data compatibility concerns, the enhancement of complexity and precision in large-scale modeling, and the optimization of algorithms and computational capabilities.

### 4.1 Multiomics data quality and format consistency

Computational biology research often involves complex datasets that exhibit multiple characteristics of ‘big data’. The term ‘big data’ encompasses four main properties that pose significant challenges for visualization: the large volume of data; the diversity of formats, data structures, and variable types; the high velocity of data retrieval, analysis, and representation; and the need to determine data validity (Rong et al., 2021; Qian et al., 2022).

Data quality and format consistency pose a significant challenge in the integration of multi-omics data due to variations stemming from diverse sources, including laboratories, platforms, and technologies. The inconsistencies can be attributed to disparities in data processing methods, experimental designs, and data acquisition and measurement approaches. Differences in genome sequencing methods, proteomics, and metabolomics measurement techniques contribute to data inconsistency, introducing measurement errors and technical variations. Instances of missing values and incomplete data further hinder data integration and analysis, potentially distorting results. Addressing these issues requires the implementation of various methods and strategies, such as data quality control, standardization, and appropriate statistical approaches (Lacchini et al., 2020).

Integrating high-throughput ‘omics’ data and multiscale modeling can reveal interactions within and between molecular scales, revealing emergent properties that cannot be solely ascribed to growth, development, reproduction, or aging at any single level in the system (Tong et al., 2020). To enable meaningful analysis, the integration of these disparate data types necessitates the meticulous implementation of data preprocessing, normalization, and integration techniques. Data visualization is essential for achieving a comprehensive understanding of metabolic networks and pathways at the systems level (Mochida et al., 2018; Watanabe et al., 2019). In response to these needs, the scientific community has focused on data visualization as a way to enhance the use of biological data for maximum effectiveness.

Validating predictions made using integrated multiomics data is challenging due to the limited availability of standard datasets. Reproducibility is also a challenge when different studies employ diverse data preprocessing or integration approaches (Dale et al., 2023). Establishing robust validation strategies, promoting data sharing, and implementing standardized analysis protocols are critical to building trust and ensuring reproducible results (Sun et al., 2022). Addressing these challenges necessitates the close collaboration of experts from various disciplines, such as biology, statistics, computer science, and bioinformatics. Progress in algorithm development, computational power, and data sharing initiatives will be instrumental in surmounting these challenges and facilitating precise predictions with integrated multiomics data (Zhou et al., 2021; Ribeiro et al., 2022; Zhan et al., 2022).

### 4.2 Reasonable use of different metabolic models and data matching challenges

The advancement of computer technology and data analysis methods has led to the application of various metabolic models in systems biology. However, these models were developed based on different plants, organ tissues, metabolites (Chalmandrier et al., 2021). Nonetheless, different models have limitations in their use. The effective use of these models and the incorporation of current large-scale multiomics data into these models pose several challenges for computational biologists and systems biologists.

The integration of big data presents challenges to mathematical models and prediction accuracy. Large data sets necessitate complex, high-dimensional mathematical models for analysis and prediction, leading to increased computational resource demands and the need for more efficient algorithms. The presence of noise, bias, or outliers in large-scale data can negatively impact the accuracy and robustness of predictive models, necessitating appropriate data cleaning and processing techniques. Moreover, the integration of diverse, structured, and unstructured data from multiple sources complicates model construction and prediction. Overcoming these challenges requires the implementation of measures during the process.

Metabolic models inherently encompass uncertainties due to the complexity of biological systems and limitations of available data. Sensitivity analysis, Monte Carlo simulations, or flux variability analysis can aid in quantifying and comprehending the uncertainties associated with model predictions (Cañas et al., 2017; Wang et al., 2019b). Genome-scale models (GEMs), kinetic models, and flux balance analysis (FBA) models have strengths and limitations. The most appropriate model depends on the specific research question and the available data. Researchers should thoroughly assess the assumptions, computational requirements, and compatibility of the model with the existing data. Precise parameterization of metabolic models is critical for accurate predictions (Tong et al., 2020; Sun et al., 2022).

**Figure 2:**
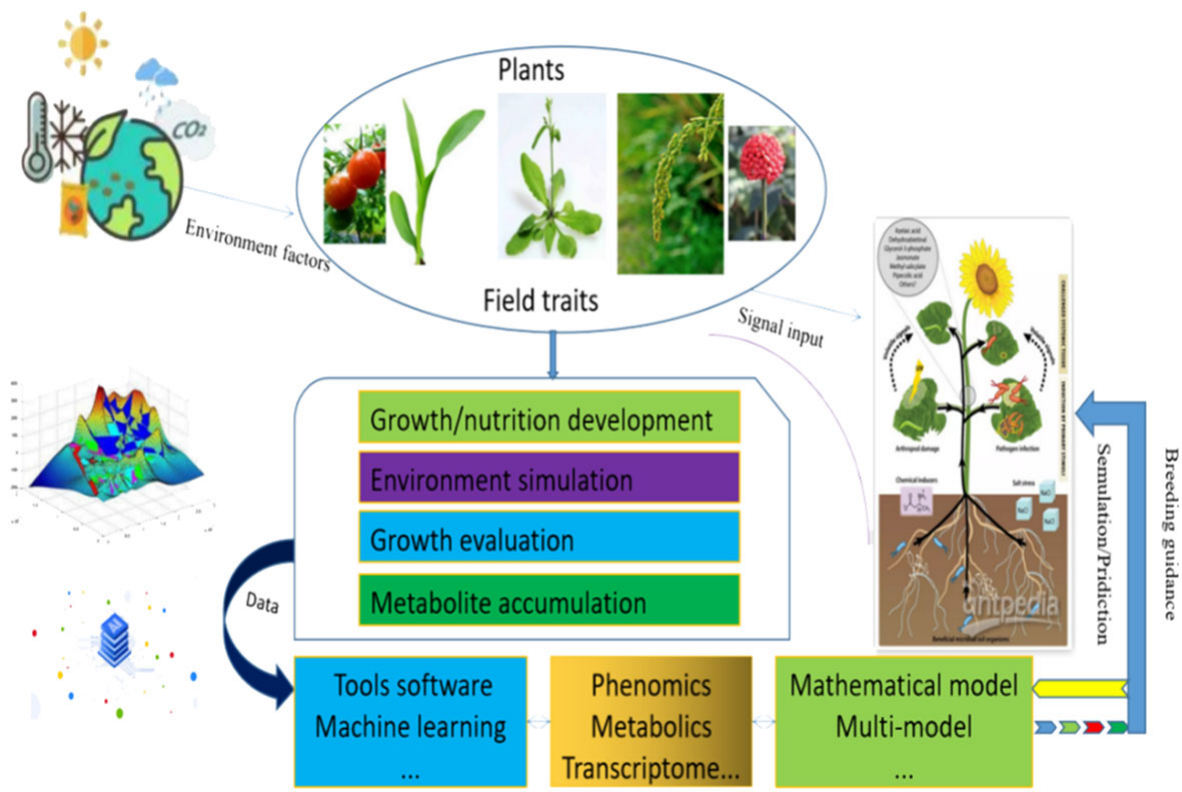
The initial assumptions of the plant production model within the framework of big data are illustrated. Plants exhibit sessile growth, rendering them vulnerable to various environmental stresses. Over extensive periods of adaptive evolution, plants have evolved specific metabolic regulatory mechanisms to sustain normal growth. Integration of artificial intelligence and big data techniques facilitates efficient extraction, analysis, and application of multi-omics data. Coupled with mathematical models for simulation and forecasting, this approach aids in optimizing agricultural practices. By utilizing models to predict plant growth under different environmental conditions, adjustments can be made to fertilization, irrigation, and plant protection strategies, tailoring them to the specific requirements of plants in particular settings. Such precision offers enhanced guidance and early detection for fostering healthy crop growth, thereby unlocking novel prospects for agricultural production and plant biology research.

Continuous improvement in integrating models and describing the metabolic processes underlying biological phenomena is essential. Urgent integration of multiscale mathematical models is necessary to guide future crop breeding and engineering, comprehend the impact of molecular-level findings on overall plant behavior, and enhance the predictive capabilities of plant and ecosystem responses in the environment. We propose employing metabolic models and machine learning techniques to predict plant production risks and offer targeted guidance for crop breeding through the effective integration of multiple omics data and the utilization of multiscale models. Plant growth biology models can be integrated with various omics data (such as genomic, transcriptomic, metabolomic, proteomic, etc.) to gain a deeper understanding of the molecular and biological mechanisms involved in plant growth processes. The following figure illustrates the schematic process of biological modeling and simulation, integrating plant growth metabolism under multiple environmental conditions with multiomics data (**Figure 2**).

### 4.3 Limitations of mathematical models in practical application

Experimental data, such as growth rates, nutrient uptake rates, and enzyme kinetics, should be utilized to calibrate the model. Validation against independent datasets is critical to confirm the model’s predictive capabilities and reliability. Even careful scientists may encounter problems during analysis. If an experiment is not well planned or executed, computational analyses, especially using toolboxes or software, often yield unreliable results (Chen et al., 2020). Although mechanistic metabolic models are rarely treated as black boxes, they can still be misused in various ways (Küken et al., 2019). The development of flexible and robust mechanisms for connecting independently developed models operating at diverse spatial and temporal scales has substantial implications for plant sciences and other fields. Biological structures display notable variations due to environmental factors and plant genetics (Spicer et al., 2017; Chalmandrier et al., 2021).

Nevertheless, the absence of mechanistic models that elucidate how a specific genotype reacts in a given environment impedes the ability to predict plant responses in untested settings. Integrative, multiscale models provide simulations that allow for the rapid examination of new scenarios, enabling the testing of the system of interest’s response to perturbations. Additionally, they aid in formulating hypotheses to guide experimental design and adopting novel technologies to obtain measurements that enhance the future applicability of plant science research (Chen et al., 2022). The complexity of plant genomes, compartmentalization of metabolic reactions, and multilevel regulation necessitate the use of dynamic metabolic models and network structure analysis through whole-genome network reconstruction to gain mechanistic insights into plant metabolic regulation (Dale et al., 2021). The time has come for a paradigm shift in plant modeling, transitional from relatively isolated research efforts to a connected community that can effectively utilize high-performance computing and a mechanistic understanding of plant processes (Smithers et al., 2021; Lu et al., 2022).

It is essential to evaluate the reliability and stability of mathematical models through validation datasets. Evaluating and forecasting the effectiveness of current breeding practices is crucial for adjusting breeding strategies to agroecological goals. Breeding efforts in recent decades, focused on optimizing individual performance, may still contribute to the performance of the focal species in mixed stands (Hu et al., 2022).

However, finding large-scale appropriate validation datasets while preventing model overfitting to training data remains challenging in agricultural production (Luca et al., 2020). Despite its advantages, constraint-based modeling still has limitations, including the requirement of a significant number of *in vivo* enzyme kinetic parameters that are currently missing for implementing enzyme constraints with the IOMA and GECKO methods (de Groot et al., 2020; Ribeiro et al., 2022). While it is possible to obtain the missing parameters from experiments or estimate them through literature searches, the process of implementing these parameters is not convenient (Noshita et al., 2022).

Plant growth and development are influenced by multiple uncertain factors and variations among individuals. Incorporating these factors into mathematical models presents a challenge because the complexity of uncertainty and diversity often surpasses the capacity of the models. Mathematical models often require adequate complexity to capture the intricacy of plant growth and development. However, this complexity can decrease the interpretability of the models, making it difficult to understand and explain the model results, thereby restricting their practical application.

## 5 Future perspective and conclusion

Over the past decade, we have observed rapid advancements in omics detection technology and the accumulation of vast amounts of biological data encompassing phenotype screening, gene sequencing, proteomics, transcriptomics, metabolomics, etc. Additionally, we have gained significant insights from metabolic models that describe life processes.

Mathematical and computational methods are becoming increasingly prevalent in the field of plant biology due to improved access to computational resources and advancements in education (Carlson and Zeng, 2023). Plant computational biology is a field that addresses this demand by bringing together experts in applied metabolic biology and computational biology who possess expertise in both metabolic and computational tools as well as their applications in plant biology (Wang et al., 2019b). We strongly encourage plant biologists who are interested in enhancing their research through computational modeling to address these challenges, recognize the scientific and specialized nature of modeling, and initiate collaborative discussions with patients.

Mathematical models play an important role in studying plant growth and development processes (Tokuda et al., 2019). The latest trend is to construct more accurate and detailed growth models to predict the growth of plants under different environmental conditions, such as the influence of factors such as light, temperature, and soil moisture on plant growth. The latest trends in mathematical modeling in plant research encompass growth models, genetic models, simulation models, and spatial models. These trends contribute to the advancement of plant science and provide foundational support for plant breeding, genetic improvement, and ecological conservation. With the advancement of technology and scientific development, mathematical modeling constantly encounters new opportunities and challenges. Consequently, our understanding of biological systems science and the underlying mechanisms behind life is continually updated, enabling us to apply this knowledge to manipulate nature.
